# Role of core promoter sequences in the mechanism of swarmer cell-specific silencing of gyrB transcription in Caulobacter crescentus

**DOI:** 10.1186/1471-2180-5-25

**Published:** 2005-05-17

**Authors:** Jennifer C England, James W Gober

**Affiliations:** 1 Department of Chemistry and Biochemistry and Molecular Biology Institute, University of California, Los Angeles Los Angeles, CA, 90095-1569, USA

## Abstract

**Background:**

Each *Caulobacter crescentus *cell division yields two distinct cell types: a flagellated swarmer cell and a non-motile stalked cell. The swarmer cell is further distinguished from the stalked cell by an inability to reinitiate DNA replication, by the physical properties of its nucleoid, and its discrete program of gene expression. Specifically, with regard to the latter feature, many of the genes involved in DNA replication are not transcribed in swarmer cells.

**Results:**

We show that for one of these genes involved in DNA replication, *gyrB*, its pattern of temporal expression depends upon an 80 base pair promoter region with strong resemblance to the *Caulobacter crescentus *σ^73 ^consensus promoter sequence; regulation does not appear to be affected by the general strength of the promoter activity, as mutations that increased its conformity with the consensus did not affect its cell-cycle expression pattern. Transcription from the *gyrB *promoter *in vitro *required only the presence of the σ^73 ^RNA polymerase (from *E. coli*) and the requisite nucleoside triphosphates, although a distinct binding activity, present in crude whole-cell extracts, formed a complex *gyrB *promoter DNA. We also assayed the effect on *gyrB *expression in strains containing mutations in either *smc *or *dps*, two genes encoding proteins that condense DNA. However we found there was no change in the temporal pattern of *gyrB *transcription in strains containing deletions in either of these genes.

**Conclusion:**

These experiments demonstrate that *gyrB *transcription does not require any auxiliary factors, suggesting that temporal regulation is not dependent upon an activator protein. Swarmer-specific silencing may not be attributable to the observed physical difference in the swarmer cell nucleoid, since mutations in either *smc *or *dps*, two genes encoding proteins that condense DNA, did not alter the temporal pattern of *gyrB *transcription in strains containing deletions in either of these genes. Rather a repressor that specifically recognizes sequences in the *gyrB *promoter region that are also probably essential for transcription, is likely to be responsible for controlling cell cycle expression.

## Background

The bacterium *Caulobacter crescentus *divides asymmetrically once during each cell cycle, yielding a sessile stalked cell and a motile swarmer cell [1,2]. These two cell types differ not only in morphology but also in their ability to replicate their genomes and to divide. The stalked cell immediately reinitiates DNA replication and cell division. The swarmer cell, in contrast, is incompetent for these two processes until it sheds its single, polar flagellum and differentiates into a stalked cell. The stalked cell then passes through several predivisional stages, during which time the genome is replicated and segregated, the newly synthesized DNA is methylated, and a new flagellum is synthesized and constructed at the pole opposite the stalk [1-4]]. The asymmetry that distinguishes the two daughter cells is founded in the predivisional cell before cell separation occurs. Asymmetric cell division depends upon several general mechanisms, including intracellular protein and/or mRNA localization, regulated phosphorylation and proteolysis, and cell-cycle dependent transcription and translation.

The inability of the *Caulobacter *swarmer cell to replicate DNA is due in part to the activity of the response regulator CtrA, which binds to sites in the origin of replication (*Cori*) [5,6]. CtrA is synthesized and phosphorylated when DNA replication is approximately half-way complete; the phosphorylated protein persists in the swarmer cell after cell division [7,8]. Phosphorylated CtrA represses transcription of DNA adjacent to the *Cori*, that is necessary for replication initiation, and also prevents binding of the DnaA initiator protein [6,9,10]. When the swarmer cell differentiates into a stalked cell, CtrA is proteolyzed and DNA replication commences [7]. In addition to its role in preventing DNA replication, CtrA is also a transcriptional activator of many genes that affect several different aspects of *Caulobacter *development. CtrA regulates transcription of its own gene, activates early genes in flagellar biogenesis, and represses *ftsZ*, the earliest known gene in cell division [5,8,11]. Interestingly, while microarray analysis has implicated CtrA in the control of approximately 25% of the cell-cycle regulated genes and it appears to directly regulate 55 operons, it does not regulate the transcription of several genes involved in DNA replication [12,13]. Thus, control of DNA replication initiation also depends on CtrA-independent transcription regulation.

Transcription of many DNA replication genes begins just before DNA replication initiates at the swarmer-to-stalked cell transition. These swarmer-cell-silenced genes include *dnaA*, *dnaN *(encoding the β subunit of DNA polymerase III), *dnaX *(γ and τ subunits of DNA polymerase III), *dnaK *(replication-initiation chaperone), and *gyrB *(B subunit of DNA gyrase) [14-18]]. The predicted promoter sequences for most of these genes align well with the published consensus sequence for σ^73 ^[19]. As the promoters of these DNA replication-associated genes appear to contain neither a CtrA binding site or any obvious elements that would suggest transcription activation in stalked cells, the mechanism responsible for their cell-cycle dependent regulation remains unknown.

In this study we analyze the role of the *gyrB *promoter in swarmer cell-specific silencing of gene expression. We map the site at which transcription of *gyrB *starts and create deletions to further delineate the promoter sequences required for temporal regulation of the gene to an 80 bp region. Using site-directed mutagenesis we further show that transcriptional silencing in swarmer cells does not appear to depend upon the overall strength of the promoter activity. *In vitro *experiments demonstrate that *gyrB *transcription does not require any auxiliary factors, suggesting that temporal regulation is not dependent upon an activator protein. However, gel-mobility shift assays demonstrate that an activity, present in *C. crescentus *crude whole-cell extracts and unrecognized by anti-RNAP antibody, is able to bind to the *gyrB *promoter. One possibility is that swarmer-specific silencing is linked to the observed difference in the physical characteristics between the swarmer cell nucleoids and stalked cell nucleoids [20-23]. Thus, a protein that affects the overall folding and structure of the nucleoid might also be regulating transcription. In this regard, we also assayed the effect on *gyrB *expression in strains containing mutations in either *smc *or *dps*, two genes encoding proteins that condense DNA. However we found there was no change in the temporal pattern of *gyrB *transcription in strains containing deletions in either of these genes.

## Results

### Mapping and deletion analysis of the gyrB promoter

**Figure 1:**
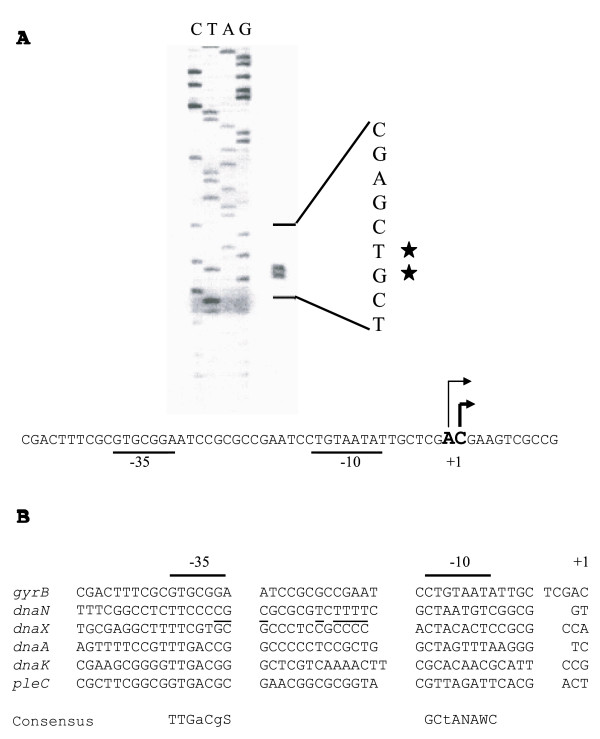
**The *gyrB *transcription start site and promoter. (A) **The transcriptional start site of *gyrB *was mapped by primer extension analysis, using an oligonucleotide primer complementary to a sequence just upstream of the translational start site. Transcription initiates at two adjacent sites, indicated by stars, approximately 170 bp upstream of the predicted start of translation. **(B) **The *gyrB *promoter was compared with other known σ^73 ^promoters, both cell-cycle regulated and constitutive; predominantly the former are shown [14-16,19,24-26]. The start sites and predicted -10 and -35 promoter elements are indicated above the sequence alignment; the published *C. crescentus *consensus sequence for genes transcribed by the common σ^73 ^RNAP holoenzyme is shown below, where S stands for G or C, W indicates A or T, and N means any nucleotide [19,26]. The lines above the *dnaX *sequence indicate the previuosly identified RRF element [24].

Previous studies of the *gyrB *gene found that transcription is silenced in swarmer cells and begins when the swarmer cell differentiates into a stalked cell [17]. These experiments also demonstrated that *gyrB *is transcribed from its own promoter and is not part of an operon, this despite the fact that the predicted start codon overlaps with the upstream *recF *stop codon [17]. Because the original *gyrB*-promoterless *lacZ *reporter fusion used in these studies contained a relatively large region of DNA upstream of the predicted start of translation, we wished to map the *gyrB *promoter to the minimal region capable of sustaining wild-type temporal regulation of the gene. Primer extension revealed two adjacent transcription start sites, separated from the translation start site by a relatively long leader sequence of approximately 170 base pairs (Fig. 1A). Note that in a separate study a slightly different start site, four base pairs further upstream, was also identified [14]. While we did not detect this start site in either the primer extension assay or in an *in vitro *transcription/primer extension experiment (see below), the alternative transcription start site was taken into consideration in subsequent mutagenesis experiments.

Based on the transcription start sites found by primer extension, alignment and comparison with several other known σ^73 ^genes allowed identification of possible -10 and -35 promoter elements of *gyrB *(Fig. 1B) [14-16,19,24-26]. The consensus is derived from σ^73 ^transcribed genes that are expressed at relatively constant levels throughout the cell cycle; expression of *gyrB*, along with other DNA-replication genes, varies during the cell cycle. In analyzing the *gyrB *promoter and surrounding DNA, we considered two broad possibilities for *gyrB *transcription regulation. *gyrB *expression might be controlled by the activity of a swarmer cell-specific factor that inhibits transcription or by a stalked cell-specific factor that activates transcription. An (A+T)-rich region, beginning approximately 60 bp upstream of the transcription start site, at first appeared to be a good candidate for a possible regulatory protein binding site. This region consists of two stretches of repeated thymidine residues and one stretch of repeated adenine residues, approximately phased with respect to each other on the DNA strand (data not shown). Such an arrangement could create a bend in the DNA, a secondary structure that is preferred by some DNA-binding proteins and that can affect transcription independent of any additional protein constituents [27,28]. Another part of the *gyrB *gene that might possibly contain a *cis*-acting regulatory site is the extremely long (170 bp), untranslated leader sequence between the transcription and translation start sites. Alternatively, *gyrB *transcription might not depend on any remote DNA elements or sites for sequence-dependent DNA binding proteins. As has been reported, the putative *gyrB *promoter appears more similar to a subset of σ^73 ^genes that mirror its temporal regulation than to those genes that are constitutively expressed throughout the cell cycle (Fig. 1B) [14,17,24,26]. This would seem to suggest that temporal regulation is dictated by the promoter itself rather than by a more distantly located DNA element.

**Figure 2:**
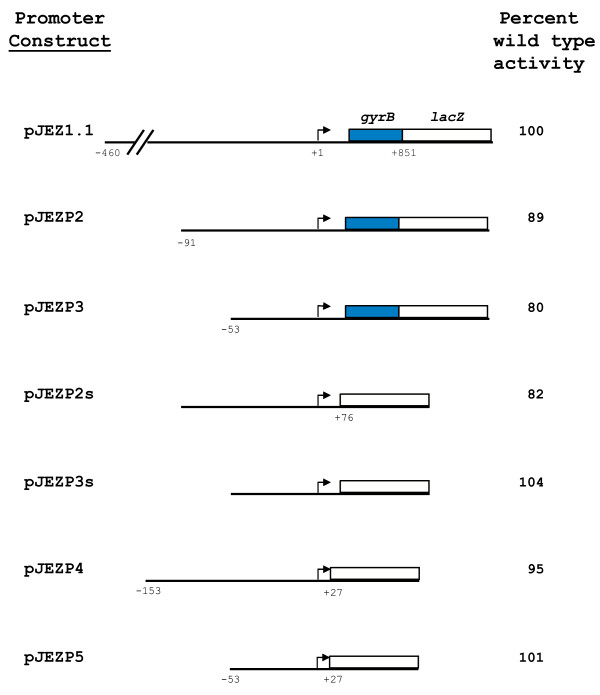
**Deletion analysis of the *gyrB *promoter region. **PCR-produced *gyrB *deletions were fused to a promoter-less *lacZ *gene in the p*lacZ/290 *vector. Transcriptional activity, measured by β-galactosidase production, is shown as a percent of wild-type (pJEZ1.1) activity (3,968 units). For each construct, the percentage is based on a mean value calculated from β-galactosidase activities of three or more different mid-log phase cultures. The base pair positions relative to the start of transcription are marked below each fusion. White boxes indicate the promoter-less *lacZ *gene and gray boxes represent the *gyrB *coding sequence.

**Figure 3:**
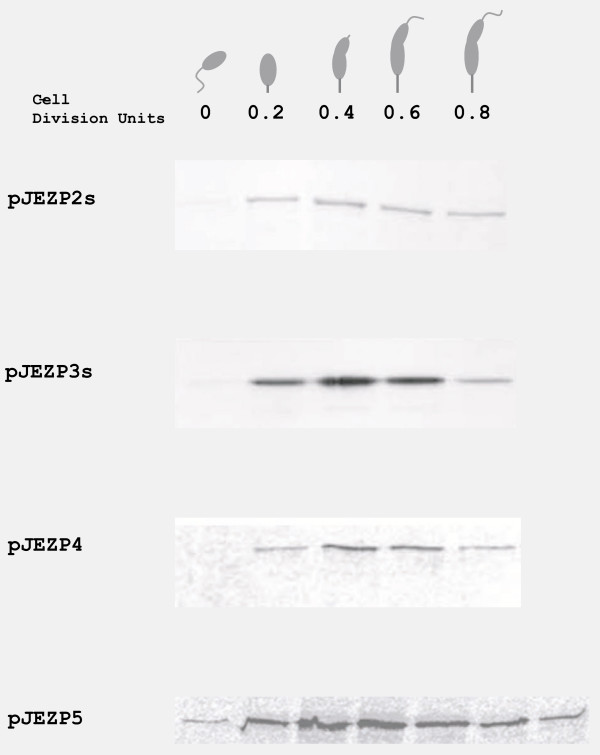
**Temporal expression patterns of the *gyrB *promoter fusions during the *C. crescentus *cell cycle. **Transcription of the *gyrB-lacZ *fusions contained on the indicated plasmids was examined during synchronized growth by immunoprecipitation of pulse-labeled β-galactosidase, as described in Material and Methods. The lanes, each revealing the β-galactosidase synthesized during one five-minute interval, are aligned beneath a diagram showing the corresponding stage in the cell cycle, as determined by light microscopy. 'Cell division units' refer to fractions of one complete cell cycle, the duration of which is approximately 140 minutes under the experimental conditions employed here. Samples were labeled every 30 minutes or every 20 minutes (pJEZP5).

In order to begin dissection of the *gyrB *promoter, we used PCR to create deletions both upstream and downstream of the core σ^73 ^promoter. We measured the transcriptional activity from these shortened promoters, each fused to a promoter-less *lacZ *gene, by β-galactosidase activity. Deletion of the upstream sequences, including the (A+T)-rich region, resulted in a slight, but not statistically significant, decrease in *gyrB *transcription (pJEZP2 and pJEZP3) (Fig. 2). A similar decrease was seen when approximately 100 bp of the untranslated leader sequence was deleted (pJEZP2s). However, wild-type transcription levels were restored by combined removal of the leader and upstream sequences (pJEZP3s, pJEZP4) and/or by removal of an additional 49 bp of the leader sequence (pJEZP5). These results indicate that the overall strength of *gyrB *promoter activity depends upon at most an 80 bp promoter region, covering -53 to +27 bp relative to the start of transcription. The temporal regulation of the deleted promoters was then investigated by immunoprecipitation of β-galactosidase produced from each construct during growth in synchronized cultures. The expression pattern of the deleted promoter fusions during the cell cycle was similar to that observed for the full-length fusion (Fig. 3). In all cases, transcription was barely detectable in swarmer cells (0 division units) followed by a marked increase in transcription at the swarmer-to-stalked cell transition (0.2 division units) and then a subsequent decrease in late predivisional cells (0.8 division units) (Fig. 3). Thus, the temporal control of the *gyrB *gene is also dictated by the core, 80 bp promoter.

### Mutagenesis of the gyrB promoter

**Figure 4:**
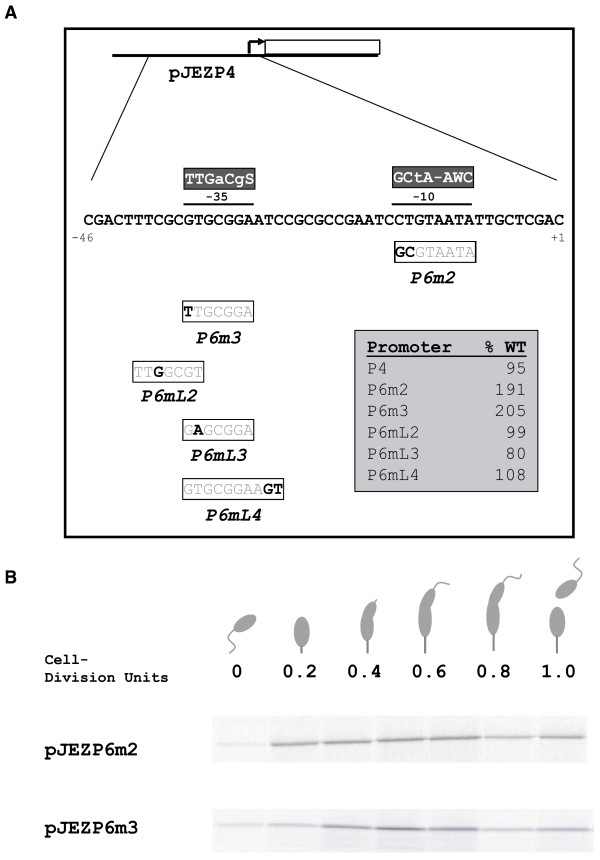
**Transcription of *gyrB *promoter mutations designed to conform to the *C. crescentus *σ^73 ^promoter consensus. (A) **The plasmid pJEZP4 contains the template promoter construct used for mutagenesis (see Fig. 3). Shown below is the DNA sequence of the promoter, with the -10 and -35 consensus regions in reverse highlight above. The mutations, shown as boxed sequences with the altered bases in bold-face, were based upon sequence alignment and were aimed at increasing the promoter's conformity with the accepted σ^73 ^consensus sequence. In a separate study, an alternative start site and subsequent alignment located the *gyrB *promoter further upstream; the mutations designed to take these data into account include an "L" in their names [14]. The transcriptional activity from the mutant promoters, as determined by β-galactosidase measurements of the corresponding *lacZ *fusions, is presented in the box in the lower right-hand corner as a percentage of wild-type (pJEZ1.1). **(B) **Temporal expression patterns of the *gyrB *promoter mutants. Transcription from the *gyrB *promoter mutants was analyzed during the *C. crescentus *cell cycle. The results for the mutants exhibiting the strongest quantitative transcription levels (pJEZP6m2 and pJEZP6m3) are shown beneath a schematic of the cell cycle that shows the corresponding developmental stage of the synchronized culture. Samples were labeled every 20 minutes.

One attractive idea to explain the temporal regulation of *gyrB *transcription is that a deviation in promoter sequences from that of the σ^73 ^consensus results in a expression levels that are somewhat weaker than that of constitutively expressed promoters. This property might make *gyrB *transcription more susceptible to a general silencing mechanism present in swarmer cells, whereas stronger σ^73^promoters would be expressed at normal levels. We investigated the importance of the basic architectural elements of the *gyrB *promoter, relative to the consensus sequence derived from constitutive σ^73 ^promoters, by site-directed mutagenesis (Fig. 4). The mutations were designed to bring the *gyrB *promoter into better agreement with the *C. crescentus *σ^73 ^consensus [19,26]. The P6m2 mutation changed the bases CT at -16 to -15 to GC, conserved at those positions in the consensus; likewise, P6m3 created a T at the -37 position. Because an alternate start site and promoter were published while these experiments were in progress, we also designed mutations based on these findings, so designated by the letter "L" in the name of the mutation [14]. The mutations P6mL2 and P6mL3 were aimed at increasing the consensus agreement of the -35 region published previously [19,26]; P6mL4 targeted a proposed 13-mer motif located between the -10 and -35 region that was also previously identified [24].

Each mutated promoter was fused to a promoterless *lacZ *gene and expression levels were quantitatively measured by β-galactosidase assays (Fig. 4A). The mutations P6m2 and P6m3 resulted in transcription levels that were twice that of the wild-type level, while the P6mL2, -3, and -4 were transcribed at normal or (P6mL3) reduced amounts. The increased promoter activity of P6m2 and P6m3 and the decreased expression of P6mL3 support the proposed promoter sequence reported here (Fig. 1) as the P6m2 and P6m3 mutations increase the agreement with the σ^73 ^consensus and while the P6mL3 mutation decreases the conformity. One possibility that could account for the increased promoter activity of the P6m2 and P6m3 mutant promoters was inappropriate expression in swarmer cells. Therefore, we wished to examine the temporal transcription patterns of these promoters in order to test this possibility (Fig. 4B). Surprisingly, transcription from the mutant promoters, though stronger, was still regulated in the wild-type manner during the cell cycle (Fig. 4B).

### Transcription of gyrB does not require an activator

**Figure 5:**
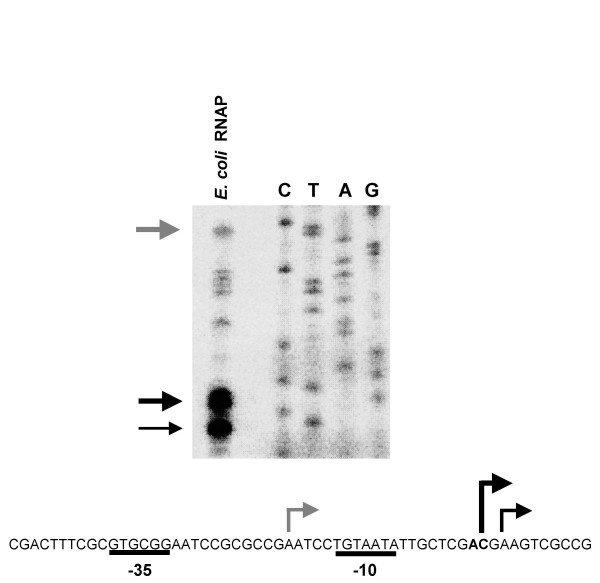
***In vitro *transcription/primer extension of *gyrB*. ***In vitro *transcription, followed by primer extension, was performed on the *gyrB *promoter template pJEKS-P3. A σ^70 ^RNAP holoenzyme preparation, obtained from *E. coli *σ^70 ^was used for transcription assays. Shown at the top are the DNA species, produced from the extended messages, next to a sequencing ladder generated from the same primer. Strong start sites are indicated by larger arrows, weaker start sites by smaller arrows. Below, is the complimentary sequence of the *gyrB *promoter, with the -10 and -35 promoter elements indicated. The major transcription start sites found by this assay are shown by upward-facing arrows, while the adjacent start sites previously found by primer extension (see Fig. 1) are shown in bold; the largest upward-facing arrow indicates a start site appearing in both assays and with both RNA polymerase species.

Because simply creating a stronger σ^73 ^promoter failed to have any effect on the temporal pattern of *gyrB *transcription, we wished to rule out the possibility that *gyrB *requires a transcriptional activator for expression within stalked cells. We employed an *in vitro *transcription assay to address this issue, using a commercially obtained preparation of *E. coli *(σ^70^) RNA polymerase (RNAP). In this experiment, transcription from a promoter on a super-coiled plasmid was assayed by primer extension. Transcription products were detected using both preparations of RNAP, indicating the lack of a requirement for an auxiliary transcriptional activation factor (Fig. 5). Of the several start sites observed, one site was present when either RNAP species was used in the *in vitro *assay (Fig. 5) as well as when RNA was obtained directly from cultured cells (see Fig. 1A). The presence of one common start site is further evidence for the correct identification of the *gyrB *promoter reported here. From this experimental result, we can conclude that the sole protein requirement for *in vitro *transcription of the *gyrB *gene is the σ^70 ^(σ^73^)RNAP holoenzyme.

### An activity, distinct from RNA polymerase, binds the gyrB promoter in vitro

**Figure 6:**
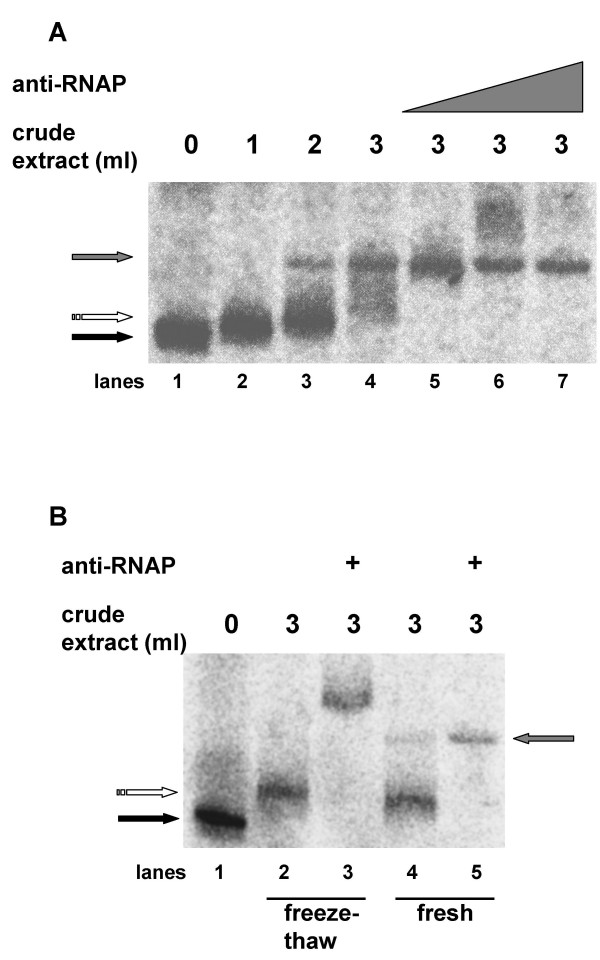
**Gel-mobility shift assays reveal a distinct, labile activity that binds to the *gyrB *promoter. (A) **A 500 base pair [^32^P]-end-labeled DNA fragment containing the *gyrB *promoter was used as a binding template for incubation with increasing amounts of fresh *C. crescentus *crude cell extract (approximately 1 to 3 mg/ml), without (lanes 1–4) or with (lanes 5–7) increasing amounts of anti-RNAP antibody. **(B) **The same binding template was incubated with 3 μl of fresh crude extract (lanes 4–5) or extract that had been previously re-frozen (lanes 2–3), with and without anti-RNAP antibody (3 μl). All binding mixtures were incubated at room temperature for 10 minutes and immediately loaded onto native 6% polyacrylamide gels. Black solid arrows indicate probe alone; gray solid arrows indicate a potential freeze-thaw-labile, shifted species; white, broken arrows indicate a binding activity that is super-shifted by anti-RNAP antibody.

Because transcription from the *gyrB *promoter did not appear to require an activator protein, it seemed likely to us that temporal regulation of the gene during the cell cycle was not attributable to stalked cell-specific transcriptional activation. An alternative possibility is that the gene is specifically repressed in swarmer cells and in the swarmer pole of the predivisional cell, possibly by a protein that binds to the promoter. We therefore decided to use gel-mobility shift assays to look for any binding activities present in *C. crescentus *crude cell extracts that interacted with the *gyrB *promoter. A very weak shift was observed when fresh crude extract was incubated with the probe (Fig. 6A, lanes 2–3, white broken arrow); this shift is apparently due to the binding of RNAP, evidenced by the further retardation of mobility when anti-RNAP antibody was added to the incubation mixture (Fig. 6A, lanes 5–7). An additional complex was seen with increasing volumes of crude extract (Fig. 6A, lanes 3–4, gray arrow). This binding activity appears to be distinct from RNAP binding as the shifted band is unaffected by the addition of anti-RNAP antibody (Fig. 6A, lanes 5–7).

Interestingly, we observed different results when we compared samples containing fresh extracts (and extracts that had been frozen only once) with those containing extracts that had been previously thawed and then refrozen. The non-RNAP binding activity was labile under conditions in which the extract had been subjected to one round of freeze-thaw (Fig. 6B, lanes 2 and 3 vs. lanes 4 and 5). Furthermore, when the extract that had been refrozen and thawed was used in the binding assay (Fig. 6B, lanes 2,3), the disappearance of the specific, labile protein band was accompanied by the appearance a new specific super-shifted band upon the addition of anti-RNAP antibody.

### The role of DNA condensing proteins in the regulation of gyrB transcription

Previous studies have shown that, in addition to being unable to initiate DNA replication, *C. crescentus *swarmer cells also differ from stalked cells in their chromosomal architecture [21-23]. These experiments revealed that isolated swarmer cell nucleoids sediment faster in a sucrose gradient than those of stalked cells. One hypothesis is that the swarmer cell nucleoid possesses a different complement of DNA 'condensing' proteins such as histone-like proteins. The gel mobility shift activity present in crude cell extracts may reflect the binding of a protein that alters the overall architecture of the swarmer cell nucleoid. Two candidates for such an activity may be either Dps or SMC. In *E. coli*, the Dps protein is induced both in stationary phase and during starvation; it is able to physically protect the nucleoid from oxidative and nuclease damage, as well as affect transcription under these conditions [29-32]. SMC (Structural Maintenance of Chromosomes), is a member of the eukaryotic SMC family [reviewed in [33-36]], large, multidomain proteins that associate with each other in an antiparallel fashion. This association occurs through long, coiled-coil domains that flank a central hinge region, resulting in protein dimers that are shaped like old-fashioned hairpins. Dimers of SMC associate with non-SMC factors to form specific complexes in eukaryotic cells, with functions in DNA organization and movement, ranging from condensation to dosage compensation to repair [33,37]. Experiments in *Bacillus subtilis *have demonstrated that chromosomal DNA was visibly decondensed in *smc *mutants [38].

The *Caulobacter dps *gene is approximately 50 to 55% identical and 60 to 65% similar to *dps *genes in other organisms, including *Burkholderia pseudomallei*, *Bordetella pertussis *and *Pseudomonas syringae *[data not shown]. Furthermore, a search of the NCBI CDD (conserved domain database) revealed significant alignment with a COG (cluster of orthologous genes) for Dps/ferritin-like DNA-binding proteins. The gene appears to be the first in a two-gene operon; the expected product of the second gene is a conserved hypothetical protein of unknown function. A *lacZ *transcription fusion of a DNA fragment spanning 600 bp upstream of the predicted *dps *translation start site was expressed at a low level (350–400 units of β-galactosidase activity) in late log-phase cultures [data not shown]. Although it has been shown to be involved in DNA protection and starvation and stationary-phase survival in other organisms, it seemed possible that Dps might be involved in developmental events specific to the swarmer cell: the results of genome-wide DNA microarray experiments showed that *dps *mRNA is maximally present in swarmer cells [13,30]. As one way to investigate a possible role for Dps in *Caulobacter *development, we engineered a strain in which 90% of the *dps *coding sequence was replaced with a gene conferring resistance to gentamicin. The strain was indistinguishable from wild-type cells in logarithmic growth and morphology, and we detected no overt aberration in swarmer-cell motility or nucleoid appearance (data not shown). Additionally, we constructed a strain with a null mutation in *smc *by disrupting the chromosomal copy of the *s m c *gene by insertion of a gene conferring spectinomycin resistance [see Methods]. The deletion was confirmed by Southern blot and by immunoblot with antibodies against SMC [data not shown].

**Figure 7:**
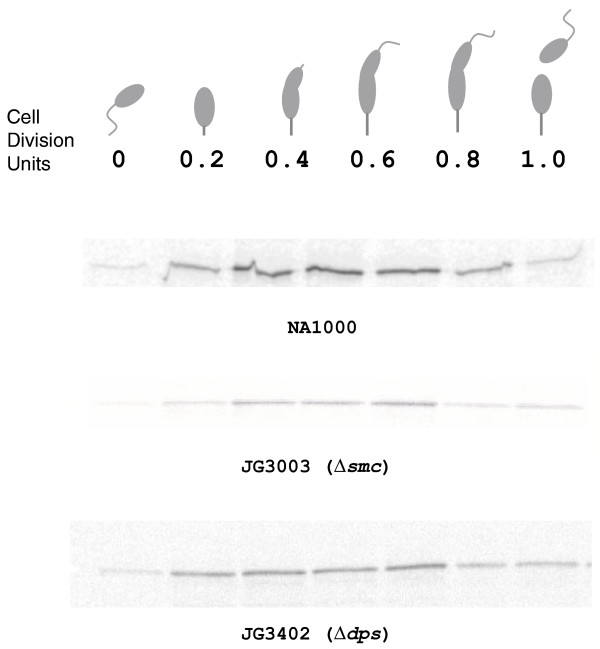
**The *gyrB *promoter is expressed in the wild-type temporal pattern in *smc *and *dps *null strains. **The plasmid pJEZP5, containing the *gyrB *promoter fused to a promoter-less copy of *lacZ*, was moved into NA1000 (wt, top), JG3003 (middle) and JG3402 (bottom) by conjugation. Transcription from the *gyrB *promoter was monitored during synchronized growth of the two strains by immunoprecipitation of β-galactosidase pulse-labeled at intervals during the cell cycle. For each strain, the protein labeled during each five-minute interval is shown beneath a diagram of the corresponding stage in the cell cycle, as determined by light microscopy.

Finally, we examined the cell-cycle transcription pattern of *gyrB *in the *smc *and *dps *mutant strains. The temporal expression pattern of a *gyrB *fused to a promoter-less *lacZ *gene was assayed by immunoprecipitation of pulse-labeled β-galactosidase in synchronized cultures of NA1000 (wild-type), JG3003 and JG3402 (Fig. 7). In both mutant strains, the *lacZ *gene was transcribed in a manner comparable to the transcription observed when the same promoter fusion was expressed in NA1000. Thus, SMC and Dps do not appear to participate in swarmer-specific transcription silencing in *Caulobacter crescentus*.

## Discussion

In *Caulobacter crescentus*, many of the genes involved in DNA replication, including *dnaN*, *dnaX*, *dnaA*, *dnaC*, *dnaK *and *gyrB*, are maximally expressed at the time that this process occurs [14-18,24,25]. Thus, transcription of these genes does not occur in swarmer cells, or in the swarmer pole of predivisional cells, and begins only when the swarmer cells differentiate into stalked cells or when DNA replication commences anew in the stalked cells at the completion of cell division. The means whereby this regulatory pattern is achieved remains unknown. In this study, we used *gyrB *as a representative gene with which to investigate the mechanism responsible for swarmer-specific transcription silencing in *C. crescentus*. As revealed by primer extension and *in vitro *transcription/primer extension assays, the transcription start site of the gene is separated from the translation start site by a fairly long leader sequence. There is apparent variation in the precise beginning of the transcript, as observed here and as reported elsewhere [14]. The lack of a precise transcription start site may be due to other factors that influence *gyrB *regulation. For example, transcription of the *gyrB *gene in *Caulobacter *is induced by relaxation of the DNA [17], and this induction is distinct from the increased expression that occurs during the swarmer-to-stalked cell transition. It is also possible that the transcription start site varies depending upon undefined regulatory cues such as growth rate and/or growth phase of the culture.

Having delineated the *gyrB *promoter to a minimal, 80 base pair region, we compared this promoter to other σ^73 ^promoters and to the published consensus sequence, taking care to explore different alignments based on the several possible start sites. The alignment shown [see Fig. 1] is that best fitting the major start site obtained by both primer extension and *in vitro *transcription/primer extension (for an alternative, see [14]). Examination of the -10 and -35 regions of the promoter, based upon our alignment, revealed that the *gyrB *promoter greatly differs from the *C. crescentus *σ^73 ^consensus sequence. This suggested an interesting possibility for a swarmer-specific silencing mechanism. We reasoned that greater deviation from the consensus sequence might make these promoters slightly inferior binding sites for the σ^73 ^RNAP, and thus slightly weaker. Such promoters might be more susceptible to a situation in which RNAP binding is partially inhibited or blocked, a possible scenario occurring in swarmer cells. Site-directed mutagenesis allowed us to test this possibility by creating versions of the *gyrB *promoter that more closely matched the consensus sequence and therefore should, in theory at least, have been transcribed at higher levels. However, while in two cases transcription from the mutated promoters was greater than from the wild-type promoter, temporal regulation of transcription was unaffected. Thus, it seems that the strength of the promoter does not intrinsically determine cell cycle regulation of *gyrB*.

The experiments presented here show that the *gyrB *promoter can be transcribed by *E. coli *σ^70 ^RNA polymerase holoenzyme in the absence of additional protein factors, suggesting the unlikelihood of temporal regulation depending solely upon an activator. However, it is possible that a stalked-cell specific transcriptional activator is required to overcome a swarmer-cell specific repression complex or DNA topological conformation. When a probe containing the *gyrB *promoter was used in gel-mobility shift assays, two binding activities, both of which are components of crude *Caulobacter *extracts, were apparent. One of these activities was loosely identified as RNA polymerase based on the ability of anti-RNAP antibody to recognize and alter the migration of this band through the gel. The other binding activity was not recognized by the antibody. This second species was vulnerable to freeze-thaw treatment, as it was only present in extract that had not been re-frozen. One might imagine a scenario in which a swarmer-specific DNA protein(s) binds to the *gyrB *promoter and interferes with the ability of the polymerase to access the DNA.

Previous experiments with another swarmer cell-repressed promoter, the *dnaX *promoter, revealed the RRF motif, that by sequence comparison is located between the -10 and -35 elements in all of the swarmer cell-silenced DNA replication gene promoters [24]. Within the RRF of the *dnaX *promoter, a C residue at position -21 appears to be involved in the temporal regulation of the gene [24]. A substitution of T at this position results in a more than 3-fold increase in transcription and, more importantly, in a loss of swarmer-specific silencing of *dnaX*. Additionally it was shown that the RRF motif is bound by an unknown species found in crude extracts [24]. The same shift was seen when DNA fragments encompassing the -10 and -35 regions of several different DNA-replication genes, including *gyrB *and *dnaX*, were used as probes in gel-mobility shift assays. It is possible that the binding activity reported here for the *gyrB *promoter is the same as that observed previously with the RRF motif. If this observed binding activity were responsible for the swarmer cell-specific silencing of *gyrB *transcription, the one might expect that swarmer cell extracts would be enriched in binding activity. However, we were unable to detect this activity in extracts derived from either isolated swarmer or stalked cells, probably owing to limitations in obtaining a sufficient quantity of material. The relevance of these DNA binding activities and a experimental definition of their binding sequences in similarly regulated promoters will need to be explored.

The condensed nature of nucleoids isolated from swarmer cells, relative to stalked cells, when subjected to centrifugation through a sucrose gradient, suggests that the two cell types differ in the proteins that affect the organization and folding of their chromosomes [20]. Growth-phase dependent variation in the relative composition of the DNA-binding protein cadre in *E. coli *has been documented [39]. A similar developmentally-regulated phenomenon might be responsible for silencing *gyrB *and other genes involved in DNA replication in *Caulobacter *swarmer cells. Proteins such as HU or H-NS bind DNA nonspecifically and have been shown to affect transcription regulation [40]. While neither protein binds to a specific sequence on the DNA, each has a preference for certain secondary DNA structures, including gaps and junctions (HU) and (A+T)-rich bends (H-NS). Of particular interest, H-NS represses the *virF *gene by binding to two sites, one overlapping the RNAP binding site in the promoter and another 200 bp upstream [41]. However, repression only occurs at temperatures below 32°C because below this temperature the DNA is more intrinsically bent, which allows interaction between H-NS bound at both sites. HU, the most abundant DNA-binding protein in bacteria, also regulates transcription. Similar to integration host factor, HU is thought to exert its influence by creating a bend in the DNA and thus affecting the activity of other, more specific transcription regulators [reviewed in [40,42,43]]. Based on searches of the published genome, we have concluded that *C. crescentus *does not possess a gene that might encode H-NS, nor does it appear to have a homolog of the closely related gene, *stpA *[42]. *C. crescentus*, does however, possess genes encoding proteins that are closely homologous to the two subunits of *E. coli *HU (HUα and HUβ). We suspect that HU is probably not involved in the regulation of the DNA replication genes since we have recently found that the abundance of both HU subunits does not change during the course of the cell cycle [unpublished observation].

In order to determine whether or not two other well-known DNA condensation proteins might affect swarmer-specific transcription, we constructed strains in which *smc *or *dps *were disrupted. There is strong evidence that the large bacterial SMC protein, very different from the small DNA-binding proteins discussed above, is involved in chromosome condensation and partitioning, similar to its eukaryotic homologs [33,36]. A deletion of the gene encoding SMC in *Bacillus subtilis *results in a temperature-sensitive strain with visibly decondensed chromosomes and defects in chromosome segregation [44,45]. Similar results have also been reported in *Caulobacter crescentus *[46]. Importantly, however, temporal transcription of *gyrB *was unchanged in the Δ*smc *mutant, indicating that SMC is not necessary for swarmer-specific transcriptional silencing.

The final target of our studies was the Dps protein. in *E. coli*, Dps protects DNA during periods of starvation or in stationary phase [31,32]. When bound to DNA, Dps forms a crystalline structure that is proposed to render the DNA physically resistant to oxidative damage as well as degradation by some nucleases [29]. Dps is also important for induced DNA protection during non-stationary phase growth, as *dps *mutants are more sensitive to hydrogen peroxide [47]. The nature of the *Caulobacter *swarmer cell (DNA replication- and cell division-incompetent) makes it conceivable that the intracellular environment may be likened to that of a cell in stationary phase or starved for nutrients. Thus, perhaps Dps plays a different role in *Caulobacter*. Supporting this hypothesis, DNA microarray experiments have revealed that the *dps *mRNA level is greatest in swarmer cells and then decreases dramatically at the swarmer-to-stalked cell transition [13]. A deletion of *dps*, however, did not have a visible effect on the *Caulobacter *cell cycle. Also, similar to the Δ*smc *strains, the temporal expression pattern of the *gyrB *promoter was unaffected in the Δ*dps *strain.

## Conclusion

In summary, these results show that a core 80 bp *gyrB *promoter sequence is sufficient to confer a cell cycle-regulated pattern of transcription. *In vitro *assays indicated that *gyrB *transcription does not require any factors in addition to RNAP. Thus, the transcription of *gyrB *is likely to be regulated by a repressor protein in swarmer cells, and not an activator protein. This repressor may be related to the DNA binding activity present in crude cell extracts that bound specifically to the *gyrB *promoter region. Deletions in the genes encoding the global DNA binding proteins, SMC and Dps had no effect on viability, growth rate or the temporal transcription pattern of *gyrB*. Thus, the results of this study do not provide definitive support for the idea that DNA-binding proteins that alter the global architecture of chromosomal DNA are involved in transcriptional repression in the *C. crescentus *swarmer cell. However, it is possible that the condensation of the swarmer-cell nucleoid is the result of the combined activity of two or more proteins and this may be important in influencing *gyrB *transcription.

## Methods

### Bacterial strains, growth conditions, and plasmid construction

Table 1 shows the strains and plasmids employed in this study. The *gyrB *fragments used in construction of promoter fusions were created by polymerase chain reaction (PCR), unless otherwise indicated, with the indicated restriction sites introduced on oligonucleotides otherwise complementary to the wild-type DNA sequence. The exception to this is the plasmid pJEZ1.1, in which the *gyrB *promoter fragment is derived from an upstream, native *Pst*I site (~-460 bp from the transcription start site), and the plasmid pJEKS-gyrB500, in which the 500 bp *gyrB *fragment is derived from the same native *Pst*I site and a native *Stu*I site at position +76 bp from the transcription start site. Generally, DNA fragments were first inserted into the plasmid pBluescript-KS+ before subsequent subcloning. The template used for the PCR reactions was purified *C. crescentus *(NA1000) chromosomal DNA; the base plasmid (p*lacZ/290*) [48] containing the fusions is a low-copy-number, plasmid, introduced into *Caulobacter *from a host *E. coli *strain (S17-1) by conjugation [49]. All *E. coli *cultures were grown at 37°C with aeration in LB broth containing (if applicable) the appropriate antibiotic [50]. *C. crescentus *strains were grown with aeration at 31°C in either peptone yeast extract (PYE) [51] or M2 minimal medium containing glucose [52].

The *dps *deletion strain was generated by the *sacB *selection method [53]. In order to accomplish this, the *dps *gene was first isolated by PCR as two separate DNA fragments that were subcloned into pBluescript KS+, reconstituting the gene with a newly-created internal MluI site. The coding region was then disrupted by introducing a gentamicin resistance cassette from pKnock GM [54] into the unique MluI site. This entire fragment, *dps2Δg*, was then subcloned into the *sacB *selection vector, pNPTS139 [M.R.K. Alley, unpublished]. The plasmid, pNPTS-dps2Δg, harbored in *E. coli *strain S17-1, was introduced into *C. crescentus *strain NA1000 by conjugation. Transconjugants were selected from PYE agar plates containing kanamycin and naladixic acid (20 μg/ml). A 2 ml culture inoculated with several transformants was grown for approximately 8 hours in PYE without selection. The culture was then diluted 1/10 and plated (0.2 ml/plate) on PYE agar containing 2.5% sucrose. One to three hundred sucrose resistant colonies were than spotted onto two different growth media: PYE sucrose plates containing gentamicin and PYE plates containing kanamycin. Sucrose-resistant, kanamycin-sensitive, gentamicin-resistant colonies where isolated for further study. The deletion of *dps *in strain pJG3402 was confirmed by PCR. The *smc *deletion strain was also isolated by this basic method except that the smc gene was disrupted with a spectinomycin resistance cassette. The *smc *deletion was confirmed by Southern blot (data not shown) and by immunoblot with anti-SMC antibodies (not shown). Following allelic exchange, the disrupted *dps *and *smc *genes were each transduced into a new wild-type *C. crescentus *strain [53].

### Cell cycle experiments

The transcription of the *lacZ *reporter fusions was assayed in cultures synchronized essentially as described previously [21]. Isolated swarmer cells were suspended in M2 glucose medium and allowed to continue growth. At various time points, 5 ml portions of growing culture were removed and the proteins were pulse-labeled for 5 minutes with ^35^S-Trans-label (ICN). Labeled β-galactosidase was immunoprecipitated as described previously [17].

### Site-directed mutagenesis and molecular biology procedures

The initial template used for mutagenesis was the 180 bp *gyrBP4 *promoter fragment. The fragment was cloned into M13-BM20 and site-directed mutagenesis was performed on single-stranded DNA isolated from *E. coli *TG-1, as previously described [55]. The mutant *gyrB *promoters were confirmed by sequencing performed according to the dideoxy chain-termination method [56]. The β-galactosidase was assayed as previously described [57,58], and the reported values represented the mean activity from at least three independently grown cultures assayed in triplicate. The standard deviation in all cases was less than 5%. All other routine molecular biology manipulations were performed as previously described [50].

### Primer extension and in vitro transcription/primer extension

Primer extension, both singly and following *in vitro *transcription, was performed as previously described [50]. The oligonucleotides (PE1: 5'-GCGTCGCCACGCGAACGC-3'; P E 2 : 5 ' – T G A G T T C G T C A G C C A G A G C – 3 ' ; B S 2 : 5 ' -GAGCAATATTACAGGATTCG-3') used in these experiments and for sequencing (data not shown) were complimentary to sequences near the 5' end of the predicted mRNA sequence and radioactively end-labeled with [γ-^32^P] ATP and T4 polynucleotide kinase [50]. In each primer extension assay, the same primer was also used for a concurrent dideoxy sequencing reaction [56]. For primer extension, total RNA was isolated from strain NA1000 (O.D. at 600 nm of 1.0) grown in M2 glucose minimal medium. Pelleted cells were resuspended in 20 mM sodium acetate, 1 mM EDTA (pH 5.4). 10% SDS was then added to a final concentration of 4%, followed by addition of an equal volume of phenol equilibrated with 20 mM sodium acetate, 1 mM EDTA (pH 5.4). Extraction, consisting of vortexing and incubation at 65°C for 10 minutes, was performed twice. The RNA was then ethanol precipitated from the aqueous phase, rinsed twice with 70% ethanol, and dried. Approximately 50 μg of the isolated RNA was used for primer extension.

*In vitro *transcription was performed as described [50]. The template was a cesium chloride gradient-purified preparation of pBluescript KS+ containing either the 900 bp *gyrBP3 *promoter fragment (see Fig. 3) or an approximately 500 bp *Pst*I/*Stu*I fragment (*gyrB500*) containing the promoter and regions further upstream (Table 1). It was transcribed by either *E. coli *RNA Polymerase σ^70 ^holoenzyme (Epicenter Technologies, Madison, WI). The resultant mRNA was then reverse transcribed from the PE2 primer, as detailed above.

### Gel mobility shift assays

The crude extracts used in these experiments were prepared from mid-log phase, 0.5-liter cultures of wild-type *Caulobacter crescentus *(NA1000). The cells were collected by centrifugation and then rinsed three to four times with HEMGK (20 mM HEPES, pH 7.6; 0.1 mM EDTA, pH 8.0; 12.5 mM MgCl2; 5% glycerol; 100 mM KCl) before being re-suspended in 4 ml of the same. PMSF in 95% ethanol was then added to 0.1% and the cells were lysed by sonication. The lysate was cleared by centrifugation at 34,500 × g in a Sorvall SS-34 rotor; the cleared lysate, containing approximately 1 to 3 mg/ml protein, was immediately frozen (-80°C) in 0.4 ml aliquots. Unless otherwise indicated, each aliquot was thawed for use only once. The DNA probe was prepared by digesting pKSgyrB500 with *Hin*dIII (a site derived from the polylinker) and *Pst*I, purifying the fragment from a 1% agarose gel and then labeling with [α-^32^P] dGTP by filling in the *Hin*dIII site with DNA polymerase I, Klenow fragment (Promega). The gel shift assays were performed essentially as described, with some minor modifications [60]. Briefly, each reaction contained 10–20 × 10^3 ^cpm of the labeled DNA probe and was incubated in Incubation Buffer (20 mM Tris-Cl, pH 7.5; 80 mM NaCl; 1 mM EDTA, pH 8.0; 0.1 mg/ml salmon-sperm DNA, sonicated) for 10 minutes at room temperature, after which loading dye was added and the samples run on a 6% non-denaturing polyacrylamide gel at 4°C. Where antibody to RNA polymerase [59] was included in the reaction, 1 to 3 μl were added and all samples were incubated an additional 5 minutes at room temperature.

## Authors' contributions

J.C.E. carried out the experimental portion of this study. J.C.E. and J.W.G. conceived of the study and wrote the manuscript.

## Figures and Tables

**Table 1:** Strains and Plasmids

**Strain**		**Reference**
*E. coli*		
		
S17-1	*Rp4-2*, *Tc::Mu*, *Km::Tn7*	[49]
TG-1	F_, *lacI*^q^, *proA*^+^*B*^+ ^*lacZ*_M15	[61]
		
*C. crescentus*		
		
NA1000 *syn-1000 *(synchronizable strain)		[22]
JG3003 *syn-1000, smc7 *(spec resistance)		[this study]
JG3402 *syn-1000, dps2 *(gent resistance)		[this study]
		
**Plasmids**		**Reference**

pBluescript-KS^+^	cloning vector	[Stratagene]
p*lacZ/290*	pRK290 derivative containing a promoterless *lacZ *gene	[48]
		
pNPTS139	*sacB *counter selection vectors; (kan^r^)	[M.R.K. Alley]
		
pJEKS- gyrB500	500 bp, *Pst*I/*Stu*I *gyrB *fragment in pBluescript-KS^+^	[this study]
		
pJEKS-P3	904 bp *Pst*I/*Hin*dIII *gyrB *promoter fragment	[this study]
pJGZ1.65	1.65 kb *Pst*I/*Eco*RI fragment in p*lacZ*/*290*, *gyrB*-*lacZ *transcriptional reporter fusion	[17]
pJEZ1.1	1.3 kb *Pst*I/*Hin*dIII fragment in p*lacZ/290*	[this study]
pJEZP2	942 bp *Pst*I/*Hin*dIII *gyrB*-*lacZ *transcriptional reporter fusion	[this study]
pJEZP3	904 bp *Pst*I/*Hin*dIII *gyrB*-*lacZ *transcriptional reporter fusion	[this study]
pJEZP2s	167 bp *Pst*I/(native)*Stu*I *gyrB*-*lacZ *transcriptional reporter fusion	[this study]
pJEZP3s	132 bp *Pst*I/(native)*Stu*I *gyrB*-*lacZ *transcriptional reporter fusion	[this study]
pJEZP4	180 bp *Pst*I/*Hin*dIII *gyrB*-*lacZ *transcriptional reporter fusion	[this study]
pJEZP5	80 bp *Pst*I/*Hin*dIII *gyrB*-*lacZ *transcriptional reporter fusion	[this study]
